# Recurrent hemichorea with different etiologies in one patient

**DOI:** 10.1093/omcr/omad006

**Published:** 2023-02-27

**Authors:** Rong Lin, Tzu-Hsien Lai, Mei-Hsiu Chen

**Affiliations:** Department of Internal Medicine, Far Eastern Memorial Hospital, New Taipei City, Taiwan; Department of Neurology, Far Eastern Memorial Hospital, New Taipei City, Taiwan; School of Medicine, National Yang Ming Chiao Tung University, Taipei City, Taiwan; Department of Internal Medicine, Far Eastern Memorial Hospital, New Taipei City, Taiwan; Department of Biomedical Engineering, Ming Chuang University, Taoyuan City, Taiwan

## Abstract

Hemichorea is a unilateral movement disorder caused by acute ischemic or hemorrhagic stroke of contralateral cerebral lesions. It is followed by hyperglycemia, and other systemic diseases. Several cases of recurrent hemichorea associated with the same etiology have been reported, but cases with different etiologies have rarely been reported. We report a case in which the patient experienced both strokes and post-stroke-related hyperglycemic hemichorea. Magnetic resonance imaging of the brain appeared different in these two episodes. Our case demonstrates the importance of evaluating every patient presented with recurrent hemichorea carefully, as the disorder may be caused by different conditions.

## INTRODUCTION

The unilateral movement disorder, hemichorea, commonly stems from acute ischemic or hemorrhagic stroke of contralateral cerebral lesions and is typically followed by hyperglycemia and other systemic diseases, such as hypoglycemia, uremia and anti-phospholipid antibody syndrome [1]. Several cases of recurrent hemichorea have been reported and associated with the same etiology [2–5], However, different etiologies could contribute to the recurrence of hemichorea in the same patient. Differential diagnosis should be made by detailed history taking, laboratory examinations and image studies. Among them, brain magnetic resonance imaging (MRI) is a helpful tool [1]. In this work, we report a case of recurrent same-side hemichorea with different etiologies diagnosed by brain MRI images.

## CASE REPORT

A 58-year-old man with a history of type 2 diabetes for 12 years was presented at our hospital 4 years ago. Because of poor compliance, the patient has not been able to achieve the target glycemic treatment goal during the past 4 years. We observed two episodes of right hemichorea resulting in similar involuntary movements but in different brain MRI images and etiologies.

### The first episode

**Figure 1:**
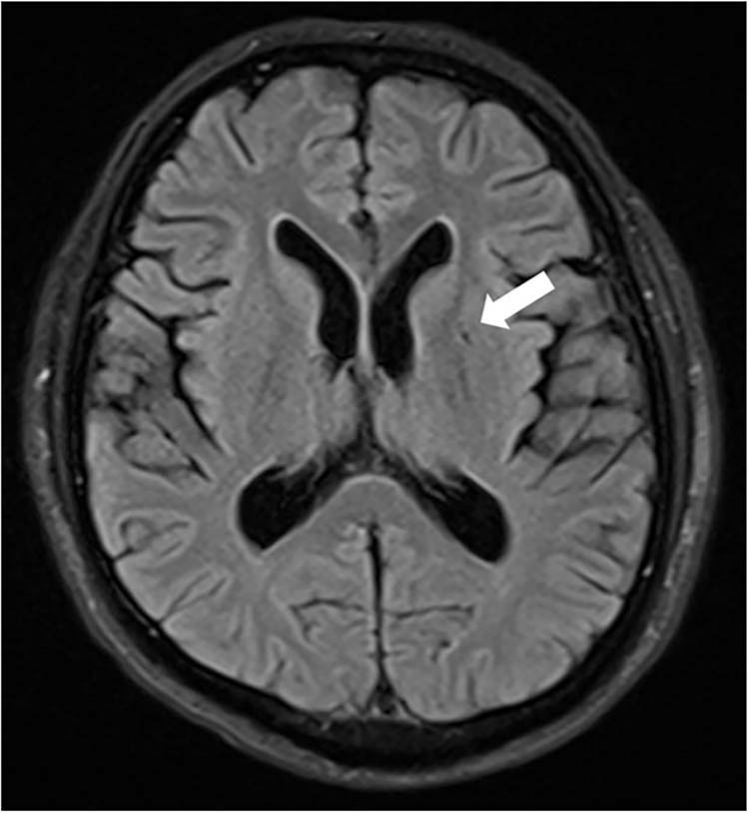
MR FLAIR image demonstrated one tiny hyperintensity lesion on basal ganglia (arrow) in patient’s first admission.

The first episode of right hemichorea brought him to our emergency room 2 weeks after a fall. Hyperglycemia (712 mg/dl) was also noted at that time. However, a computed tomography (CT) scan of the brain disclosed no specific findings. He was admitted and an MRI scan of the brain showed an old lacunar infarct at the left basal ganglion (Fig. 1). His right hemichorea was resolved the day after admission without any treatment of the disorder. He had no neurologic deficit. Basal bolus insulin therapy was given for blood sugar control, and aspirin was given to prevent recurrent stroke after discharge.

### The second episode

**Figure 2:**
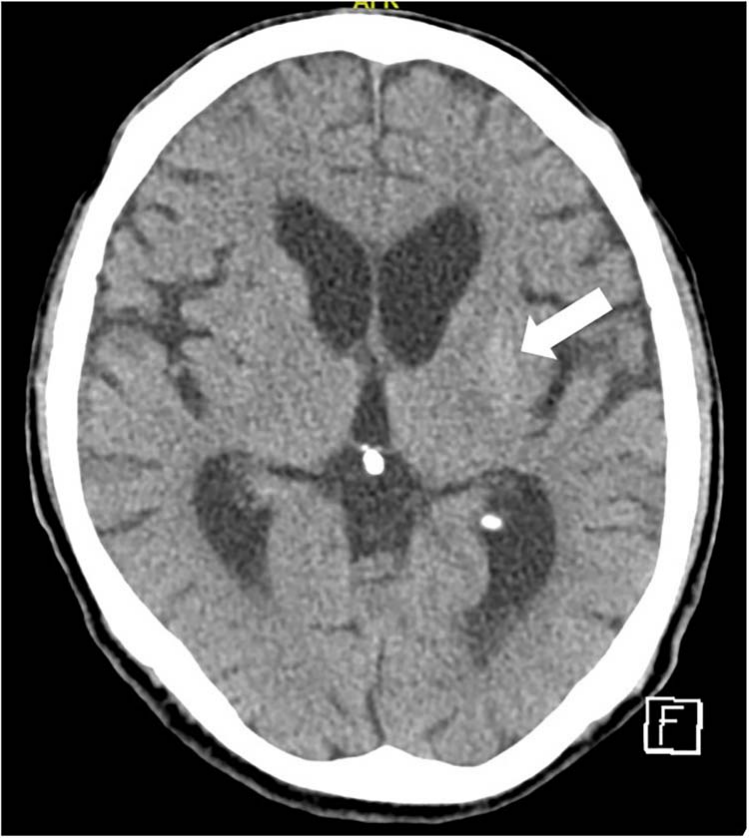
Brain CT scan showed increased density in the left basal ganglia (arrow) during patient’s second admission.

**Figure 3:**
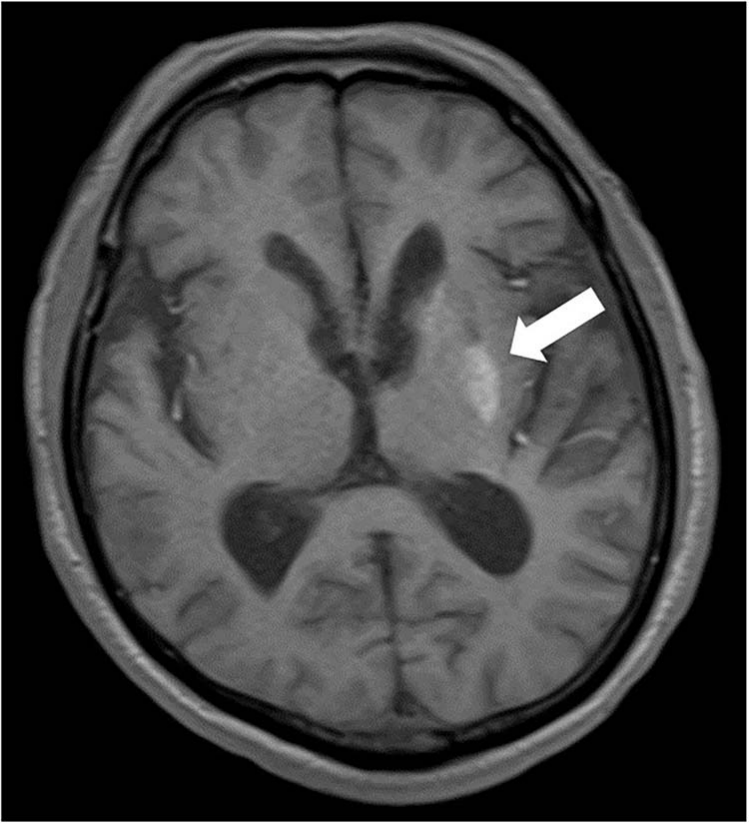
T1 MRI showed hyperintensity in the left basal ganglia (arrow) during patient’s second admission.

After 4 years of follow-ups in the outpatient department, the patient’s compliance was still poor. He was hospitalized because of involuntary movement in his right upper limb and right jaw for 2 weeks. The involuntary movement subsided when he fell asleep. The neurological examination showed that muscle power and sensation of bilateral limbs were intact. His blood sugar was high (671 mg/dl) without ketoacidosis. The serum creatinine level was normal, and the HbA1c was 17.3%. A CT scan of brain showed increased density in the left basal ganglia (Fig. 2). Further MRI scan was also performed to exclude acute stroke and disclosed an increased signal intensity on the T1-weighted image in left basal ganglion (Fig. 3), which was compatible with hyperglycemic hemichorea. Initially, we prescribed Clonazepam and haloperidol for the hemichorea and basal bolus insulin for the hyperglycemia. His blood sugar improved dramatically, but the hemichorea only improved a little after 10 days of hospitalization. He was discharged with Clonazepam 0.5 mg QD for the hemichorea, and basal insulin and oral anti-diabetic agents for glycemic control. Two weeks later at an outpatient visit, his hemichorea improved gradually because the blood sugar was better controlled.

## DISCUSSION

We reported a case of recurrent hemichorea in a 58-year-old man exhibiting different etiologies demonstrated by specific brain MRI findings. The most common cause of hemichorea is acute ischemic or hemorrhagic stroke, and the second is hyperglycemic hemichorea. Other causes including hypoglycemia, Sydenham’s disease, uremia and anti-phospholipid antibody syndrome have also been reported. These different etiologies of hemichorea result in different brain imaging appearance [1]. Typically, in hyperglycemic hemichorea, CT scans show hyperdense lesions at the contralateral basal ganglia and T1 MRI shows hyperintensity [6, 7]. Both the CT and T1 MRI of our patient showed similar features. In an acute stroke, the MRI demonstrates lesions with hyperintensity in diffusion-weighted images and with hypointensity in apparent diffusion coefficient maps. In an old ischemic stroke, MR fluid-attenuated inversion recovery (FLAIR) images demonstrate hyperintensity [8]. In hypoglycemia, T2 hyperintensity was noted at the basal ganglia [9]. In uremia, T2 hyperintensity lesions with edematous and a rim enhancement were noted [10]. Therefore, the MRI is a useful tool for the differential diagnosis of the causes of hemichorea.

In addition, hyperglycemic hemichorea is a rare neurologic complication of poorly controlled diabetes, which was first described by Bedwell in 1960 [11]. Most cases were old Asian women. The prognosis of hyperglycemic hemichorea was good and rare recurrences were reported after blood sugar was better controlled. The mean recovery days of hyperglycemic hemichorea is about 69 days [12], according to a recent report in Korea. Blood sugar control is key for the recovery of hyperglycemic hemichorea and neuroimage findings on follow-up MRIs are usually reversible as well [13, 14]. In our patient, recurrent hemichorea with different etiologies was diagnosed primarily based on different neuroimaging findings, but also based on differences in the durations of the courses. The duration of the hemichorea was short for the first episode and diagnosed to be stroke related. The duration of the hemichorea was longer for the second episode and was diagnosed to be hyperglycemia related. This could be caused by the different etiologies or by hyperglycemic stress superimposed on the same atherosclerotic lesion.
